# Mitochondrial Cholesterol in Alzheimer's Disease and Niemann–Pick Type C Disease

**DOI:** 10.3389/fneur.2019.01168

**Published:** 2019-11-07

**Authors:** Sandra Torres, Carmen M. García-Ruiz, Jose C. Fernandez-Checa

**Affiliations:** ^1^Department of Cell Death and Proliferation, Instituto de Investigaciones Biomédicas de Barcelona, Consejo Superior de Investigaciones Científicas, Barcelona, Spain; ^2^Liver Unit and Hospital Clinc I Provincial, Centro de Investigación Biomédica en Red (CIBEREHD), Institut d'Investigacions Biomèdiques August Pi i Sunyer, Barcelona, Spain; ^3^Southern California Research Center for ALDP and Cirrhosis, Los Angeles, CA, United States

**Keywords:** cholesterol, mitochondria, lysosomal disorders, sphingolipids, acid ceramidase

## Abstract

Mitochondrial dysfunction has been recognized as a key player in neurodegenerative diseases, including Alzheimer's disease (AD) and Niemann–Pick type C (NPC) disease. While the pathogenesis of both diseases is different, disruption of intracellular cholesterol trafficking has emerged as a common feature of both AD and NPC disease. Nutritional or genetic mitochondrial cholesterol accumulation sensitizes neurons to Aβ-mediated neurotoxicity *in vitro* and promotes cognitive decline in AD models. In addition to the primary accumulation of cholesterol and sphingolipids in lysosomes, NPC disease is also characterized by an increase in mitochondrial cholesterol levels in affected organs, predominantly in brain and liver. In both diseases, mitochondrial cholesterol accumulation disrupts membrane physical properties and restricts the transport of glutathione into mitochondrial matrix, thus impairing the mitochondrial antioxidant defense strategy. The underlying mechanisms leading to mitochondrial cholesterol accumulation in AD and NPC diseases are not fully understood. In the present manuscript, we discuss evidence for the potential role of StARD1 in promoting the trafficking of cholesterol to mitochondria in AD and NPC, whose upregulation involves an endoplasmic reticulum stress and a decrease in acid ceramidase expression, respectively. These findings imply that targeting StARD1 or boosting the mitochondrial antioxidant defense may emerge as a promising approach for both AD and NPC disease.

## Introduction

Neurodegenerative diseases encompass a wide range of neurological disorders caused by different causes, most notably genetic mutations in specific genes. Alzheimer's disease (AD) is one of the most prevalent neurodegenerative diseases in which the progressive loss of neurons is associated with the upregulation of peptides and activation of proteins, such as amyloid beta (Aβ) or tau phosphorylation, that trigger specific signaling pathways that ultimately contribute to the progression of the disease. In addition, accumulation of other cellular components, such as specific types of lipids, can cause neuronal death and mitochondrial dysfunction in the brain and in peripheral organs. The role of mitochondrial dysfunction in neurodegenerative diseases remains to be fully elucidated. Recent studies have provided evidence that alterations in lipid metabolism can have a deleterious impact on mitochondrial function, which can contribute to the progression not only of AD but also of the lysosomal storage disorder Niemann–Pick type C (NPC) disease, a neurovisceral disorder primarily characterized by the accumulation of lipids in intracellular organelles, most predominantly in lysosomes. Here, we briefly summarize evidence indicating that increased cholesterol trafficking to mitochondria has emerged as a putative key player in AD and NPC disease through the disruption of mitochondrial routine performance, leading to oxidative stress and cell death.

## Mitochondrial Cholesterol Trafficking in Neurodegenerative Diseases

Cholesterol is an essential component of membrane bilayers, which determines their physico-chemical and functional properties. Cholesterol is particularly enriched in the brain, where it regulates key biological functions, such as signal transduction pathways, myelin formation, and synaptogenesis (1). In the central nervous system (CNS), cholesterol is synthesized *de novo* from acetyl-CoA in the mevalonate pathway in the endoplasmic reticulum (ER) (2). Since cholesterol does not cross the blood–brain barrier (BBB), to ensure steady-state turnover, cholesterol synthesis is matched by its metabolism to 24S-hydroxycholesterol (24-OHC), which crosses the BBB and is delivered to peripheral organs and hence constitutes an intrinsic mechanism to prevent cholesterol accumulation in the brain. During the perinatal period, cholesterol accumulation in the brain is mainly determined by oligodendrocytes due to their key role in myelination, while neurons synthesize their own cholesterol *de novo* needed for neuronal plasticity and function (3). In the adult life, however, the rate of *de novo* cholesterol synthesis declines, forcing neurons to acquire cholesterol from a cross-talk between neurons and astrocytes (1, 4). Although the regulation of cholesterol homeostasis in brain diseases has been reviewed elsewhere (2), here we discuss the specific role of mitochondrial cholesterol in neurodegenerative diseases.

While much of what it is known about the trafficking of cholesterol into mitochondria has been elucidated in the context of steroidogenesis, the relevance of mitochondrial cholesterol in neurodegeneration has been less recognized. Emerging evidence indicates that mitochondrial cholesterol loading can influence mitochondrial function independently of its conversion to pregnenolone or oxysterols, arising as a key factor in the pathology of several neurological diseases associated with mitochondrial dysfunction, such as AD and NPC disease (5). Although the physiological levels of cholesterol in mitochondrial membranes are low compared to other membrane bilayers (e.g., plasma membrane), the limited content of mitochondrial cholesterol is essential for the maintenance of mitochondrial membrane physical properties and synthesis of neurosteroids. The key regulatory enzymes responsible for steroid synthesis in the CNS include the cytochrome P450 side-chain cleavage (P450scc), and the steroidogenic acute regulatory protein StARD1, the founder member of a family of lipid transporting proteins that contain StAR-related lipid transfer (StART) domains. The rate-limiting step in the synthesis of neurosteroids is the availability of mitochondrial cholesterol in the mitochondrial inner membrane (MIM) for metabolism by P450scc. Cholesterol is imported to mitochondrial membranes by the action of lipid transfer multiprotein complex acting at membrane contact sites. StARD1 plays an essential role in the transfer of cholesterol to the MIM, as inferred from the outcome of mice with global StARD1 deletion, which undergo a lethal adrenal lipoid hyperplasia, indicating that other members of the StAR family cannot replace its function in intramitochondrial cholesterol trafficking (6, 7). StARD1 contains a mitochondrial localization sequence and a steroid-binding domain. The exact location of StARD1 in mitochondria has been much discussed, but among StARD1 forms, the 30 kDa phosphorylated form has been described to be localized on the MIM (8). MLN64 (also known as StARD3) provides cholesterol to the mitochondrial outer membrane (MOM) from endosomes (5, 9) and together with StARD1 work in tandem in the net import of cholesterol to the MIM for metabolism.

**Figure 1:**
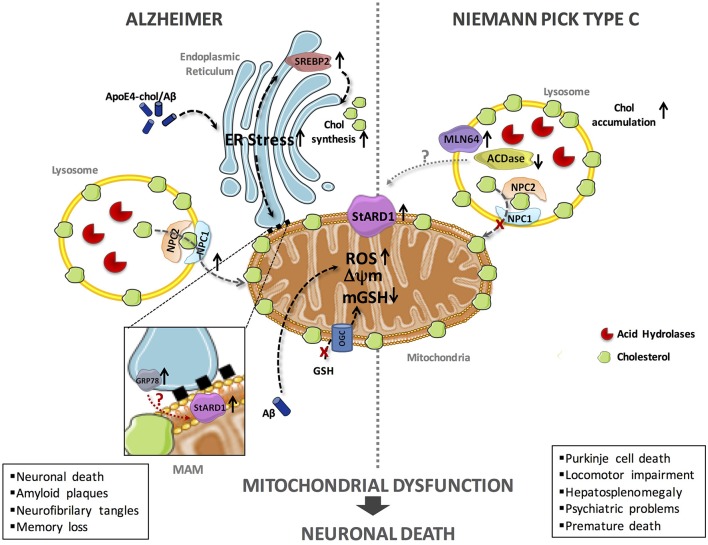
Schematic representation of the putative mechanisms of StARD1 upregulation in AD and NPC. Mitochondrial cholesterol accumulation and subsequent mGSH depletion is a common feature of AD and NPC and its correlates with increased expression of StARD1. Since StARD1 is critical for mitochondrial cholesterol trafficking, it is conceivable that StARD1 is located in the MAM interface between ER and mitochondria. While in AD, ER stress by Aβ may contribute to StARD1 upregulation, this event does not seem to play a role in NPC. Given the repression of StARD1 by ACDase, we propose that ACDase downregulation may contribute to StARD1 induction in NPC disease, which requires further investigation. In both diseases, mitochondrial cholesterol accumulation disrupts membrane physical properties and impairs the transport of GSH into mitochondria, via the 2-oxoglutarate carrier (OGC), disrupting the mitochondrial antioxidant defense and subsequent oxidative stress. Chol, cholesterol; mGSH, mitochondrial glutathione; ROS, reactive oxygen species.

In the last decade, significant progress has been made on the impact of cholesterol accumulation in mitochondrial function and routine performance in AD and NPC disease using genetic mouse models, such as the APP/PSEN1 transgenic mice overexpressing SREPB-2 (APP/PSEN1/SREBP2) and the Npc1^−/−^ knockout mice (10–14). While much of the deleterious effects of mitochondrial cholesterol accumulation in both diseases is accounted for by the depletion of mitochondrial GSH (mGSH), unfortunately the underlying mechanisms whereby cholesterol accumulates in mitochondria in AD and NPC are not fully understood. Our hypothesis posits that StARD1 is a critical player in mitochondrial cholesterol loading and hence emerges as a putative novel target for intervention in both diseases (Figure 1).

## Intracellular Cholesterol Homeostasis and Mitochondrial Function in AD

AD is one of the most common neurodegenerative disorders in older adults. The pathological hallmark of AD is the cognitive impairment and associated dementia due to neuronal death caused in part by the accumulation of amyloid plaques in the cortex and hippocampus (15–18). Currently, there is no cure for AD, which reflects our incomplete understanding of AD pathogenesis. AD is a multifactorial disease and several players contribute to its progression, including the disruption of cholesterol homeostasis. In this regard, epidemiological findings showed that hypercholesterolemia is a major risk factor for AD development (19). However, in spite of the association between hypercholesterolemia and AD, the role of cholesterol in AD is controversial and not fully understood. In this regard, a body of literature supports a link between increased cholesterol levels in the brain with the progression of AD. For instance, the specific presence of the enzymes involved in the generation of toxic Aβ peptides in lipid rafts, specific domains of membrane bilayers highly enriched in cholesterol, provides a strong association between high cholesterol levels with Aβ generation and AD development (20, 21). Moreover, in the CNS, cholesterol is transported between different cell types by a multicellular trafficking process largely regulated by ApoE (22–24). Consistent with its function in cholesterol trafficking within the brain, ApoE polymorphisms, particularly APOEε4 allele, have emerged as a risk for AD development and correlate with higher levels of Aβ in the serum (25, 26). Moreover, experimental models fed diets enriched in cholesterol have been shown to develop AD-like pathology [(27); reviewed in (28)]. Quite interestingly, besides the association between the increase in cholesterol levels with AD, there is also evidence indicating that low cholesterol levels in the brain can contribute to the AD progression (2). This inverse relationship is of particular significance during aging, as low levels of cholesterol in hippocampus are characteristic of the aged human brain (29). Furthermore, as cholesterol metabolism in the brain to 24-OHC by the action of CYP46A1 represents a unique mechanism to control brain cholesterol homeostasis, CYP46A1 polymorphisms correlate with lower brain cholesterol levels and increased risk of AD (30, 31). In addition, hippocampal cholesterol loss has been shown to contribute to the poor cognition in old rodents, and hence, cholesterol replenishment in aging animals improves hippocampal-dependent learning and memory (32).

In the early stage of AD development, mitochondria undergo significant functional deficits, which correlate with accumulation of neurotoxic Aβ (33–36). Interestingly, it has been shown that Aβ can target mitochondria to stimulate ROS generation, thus contributing to Aβ toxicity in neurons (37, 38). Immunoelectron microscopy analysis indicated the association of APP with mitochondrial protein translocation components, TOM20 and TIM23, which correlated with decreased import of respiratory chain subunits, lower cytochrome oxidase activity, and increased ROS generation (39). Moreover, functional complexes with γ-secretase activity have been found in mitochondria while insulin-degrading enzyme (IDE), which is known to contribute to Aβ removal, can be targeted to mitochondria (40, 41). Thus, mitochondrial dysfunction is associated with increased Aβ generation and AD progression.

In line with the potential relevance linking intracellular cholesterol to mitochondrial dysfunction in AD, mitochondrial cholesterol enrichment has been shown to sensitize to Aβ-mediated neurotoxicity through depletion of mGSH levels (10). Moreover, APP/PS1/SREBP-2 mice, which exhibit an early mitochondrial cholesterol loading and mGSH depletion, exhibited an accelerated β-secretase activation, Aβ accumulation, and cognitive decline compared to APP/PS1 mice (11), further supporting the correlation between mitochondrial cholesterol accumulation and the subsequent mGSH depletion in AD. Consequently, *in vivo* treatment of APP/PS1/SREBP-2 mice with the cell-permeable GSH ethyl ester, which restored mGSH levels, attenuated synaptic degeneration and improved cognition, suggesting that therapeutic strategy to prevent mitochondrial cholesterol accumulation or the restoration of mGSH levels may represent a relevant approach in the treatment of AD. Further evidence indicated that ER stress acts as a link between Aβ generation and cholesterol upregulation and subsequent mitochondrial cholesterol trafficking due to increased expression of StARD1 (12). Furthermore, administration of chemical chaperones that prevent ER stress ameliorates StARD1 upregulation and cognitive decline in APP/PS1/SREBP-2 mice.

In line with this sequence of events, recent findings provided evidence that StARD1 is an ER stress target gene, as tunicamycin-mediated ER stress induces StARD1 upregulation in primary hepatocytes that was prevented by tauroursodeoxycholic acid (42). Additional evidence linking ER stress and StARD1 upregulation derived from studies on acetaminophen (APAP) hepatotoxicity in which APAP-induced ER stress causally led to StARD1 induction that primed to APAP-induced liver injury (43). Thus, these studies provide strong evidence to support the hypothesis that Aβ-induced ER stress may be a key mechanism for AD pathology by promoting increased brain cholesterol content as a result of enhanced SREBP-2 processing, while stimulating cholesterol trafficking to mitochondria via StARD1 upregulation. Indeed, it has been reported that in the early stage of AD development, Aβ-induced ER stress is indirectly involved as an effector of Aβ-mediated neurotoxicity (44). Furthermore, AD human brains exhibit evidence for increased ER stress markers accompanied by APP accumulation and activation of β-secretase (45). In addition, Aβ peptides are reported to cause alterations in mitochondria-associated membranes (MAMs) (46, 47). In this regard, MAMs act like ER/mitochondria contact sites, transferring stress signals from the ER to mitochondria during the early adaptive phases of ER stress and in the regulation of steroidognesis (Figure 1) (48). In addition, different studies have shown a relationship between ER chaperones and StARD1 in MAMs that correlates with an increase in expression of GRP78 in AD patients (45, 49). In line with these findings, elevated STARD1 levels have been reported in the cytoplasm of hippocampal pyramidal neurons from brain samples of AD patients (50), suggesting a mechanistic link between StARD1 and mitochondrial cholesterol loading in human AD. Further work will be needed to critically establish a cause-and-effect relationship between StARD1 upregulation and the mitochondrial cholesterol accumulation and its contribution to AD, which will require the generation of cell-type-specific StARD1 deletion models in brain to examine the sensitivity to AD pathology (Garcia-Ruiz et al., manuscript in preparation).

## Mitochondrial Cholesterol Accumulation in NPC Disease

Lysosomal lipid accumulation is the hallmark of NPC disease, which is characterized by neuronal and visceral symptoms, spleen dysfunction, hepatosplenomegaly, deficits in motor coordination, and premature death (51, 52). NPC disease is caused by mutations in genes encoding NPC1 and NPC2, two lysosomal-resident proteins responsible for the egress of cholesterol from lysosomes to cytosol. Most NPC cases are due to loss of function of NPC1, and consequently, mice with NPC1 deletion *(Npc1*^−/−^ knockout mice) reproduces many of the deficits seen in NPC patients, including the neurological symptoms, ataxia by 6–7 weeks of age, and reduced maximal life span to about 10–12 weeks (52, 53). Consistent with the crucial role of NPC1/2 in intracellular cholesterol trafficking, the primary biochemical feature of NPC disease is the accumulation of cholesterol in lysosomes. However, due to the mutual regulation of cholesterol and sphingolipids to maintain a constant ratio in membrane bilayers, which is crucial for the maintenance of their physical properties, the increase of lysosomal cholesterol loading in NPC disease is accompanied by accumulation of specific sphingolipids species (54–56). In addition to the accumulation of cholesterol/sphingolipids in lysosomes, cholesterol also has been reported to accumulate in mitochondria in affected organs from Npc1^−/−^ mice, particularly liver and brain and in fibroblasts from NPC patients (57, 58). As recent findings have demonstrated that diet-induced mitochondrial cholesterol enrichment impairs mitochondrial routine performance and disrupts the assembly of respiratory supercomplexes (59), the increase in mitochondrial cholesterol seen in NPC models can contribute to the reported mitochondrial dysfunction and subsequent oxidative stress associated with NPC disease, which is largely due to mGSH depletion and subsequent disruption of mitochondrial antioxidant defense (10, 57, 58, 60–63). In line with these findings, defective ATPase activity has been reported in brain mitochondria from Npc1^−/−^ mice, and this outcome has been causally linked to cholesterol accumulation in mitochondrial membranes as its extraction with methyl-β-cyclodextrine (βCD) restored ATP activity (57). Moreover, mGSH replenishment with GSH ethyl ester (GSHEE) in cerebellum of Npc1^−/−^ mice was able to reverse mitochondrial dysfunction and improve oxidative phosphorylation. GSHEE treatment enhanced neurological performance and motor activity and, more importantly, resulted in increased median survival and maximum life span of NPC1 null mice, similar to treatment with βCD (13). However, combination of GSHEE with βCD had no additive effects, suggesting that both agents act in a common pathway affecting mitochondrial function. Furthermore, liver samples from 8 week-old NPC null mice exhibited increased protein carbonylation, decreased ATP levels, and activated caspase 3 activity, whereas isolated mitochondria revealed increased MitoSox fluorescence and reduced mitochondrial membrane potential, effects that were reversed by GSHEE therapy. Liver injury, inflammatory foci, and hepatosplenomegaly increased in liver of Npc1^−/−^ mice, and these signs of liver disease were attenuated by GSH-EE treatment. In contrast to the therapeutic effects of GSHEE, N-acetylcysteine (NAC) did not restore mGSH and failed to improve NPC pathology, although NAC was effective in increasing cytosol GSH pool. These findings illustrate the relevance of mGSH depletion in NPC disease and the need to implement specific strategies to bypass the block of GSH transport in mitochondria imposed by the accumulation of cholesterol and subsequent decrease in membrane fluidity.

As mitophagy stands as a specific mechanism to maintain mitochondrial quality control, the increase in lysosomal cholesterol, which has been shown to impair mitophagy, could contribute to the perpetuation of mitochondrial dysfunction in NPC disease due to impaired mitochondrial turnover, as illustrated in drug-induced liver injury (64). Besides the impact in quality control, recent findings have provided evidence for impaired mitochondrial biogenesis in NPC disease by a mechanism involving transcriptional repression of mitochondrial biogenesis (65). This outcome is mediated specifically by the transcription factors KLF2 and ETV1. Both are induced in NPC cells and their silencing restored mitochondrial biogenesis. Increased expression of ETV1 is regulated by KLF2, while the increase in KLF2 levels is caused by impaired signaling downstream of sphingosine-1-phosphate receptor 1, which normally represses KLF2 (65). Quite intriguingly, as mitochondrial respiratory chain deficiency regulates lysosomal homeostasis and hydrolysis (66), it is tempting to speculate that mitochondrial cholesterol-mediated dysfunction and lysosomal cholesterol accumulation engage in a mutual regulatory cycle that is of relevance to NPC disease.

Although mitochondrial cholesterol is recognized to contribute to mitochondrial dysfunction and has emerged as a putative player in NPC pathogenesis, the molecular mechanism involved in the stimulated trafficking of cholesterol to mitochondria remains poorly understood. In this regard, increased expression of StARD3 (MLN64) has been reported in NPC cells, which correlated with enhanced cholesterol accumulation in mitochondria and mitochondrial depolarization (63). In parallel with these observations, StARD1 upregulation has been observed in affected organs of Npc1^−/−^ mice and in fibroblasts from NPC patients (67). Since StARD3 is thought to transfer cholesterol from endosomes to the MOM, the increased expression of StARD1/StARD3 suggests that both proteins work together in the intramitochondrial trafficking of cholesterol, with StARD1 playing a key role in the transfer to MIM. Consistent with this possibility, hepatocyte-specific StARD1 deletion has been shown to prevent cholesterol accumulation in MIM and protect against APAP-mediated liver failure despite unchanged MLN64 expression (43). Thus, in view of the crucial role of StARD1 in mediating mitochondrial cholesterol loading, understanding the molecular mechanisms involved in StARD1 induction may be of relevance for NPC pathogenesis and could emerge as a druggable target for the treatment of NPC disease.

## Discussion

Although current evidence indicates that mitochondrial cholesterol emerges as a common event in both AD and NPC, the molecular mechanisms contributing to its accumulation in mitochondria are not well-understood. In both models, we observed increased expression of StARD1 and enhanced MLN64 expression (70–90%) (12, 63, 67). In spite of the shared upregulation of StARD1 in AD and NPC disease, the putative mechanisms underlying its induction appear to be different in both diseases (Figure 1). In this regard, although ER stress is known to induce the upregulation of StARD1 and AD is characterized by ER stress, there is no evidence for induction of ER stress markers in NPC (68), dissociating the relationship between ER stress and StARD1 upregulation in NPC and AD. Alternatively to the onset of ER stress, we hypothesized that acid ceramidase (ACDase) may be involved in the upregulation of StARD1, as its expression is decreased in liver and brain of Npc1^−/−^ mice (67). As ACDase has been shown to repress StARD1 expression through binding to the nuclear receptor steroidogenic factor-1 (69), it remains to be established whether decreased ACDase in NPC disease contributes to the upregulation of StARD1 and subsequent mitochondrial cholesterol accumulation. Thus, based on these findings, StARD1 upregulation in AD and NPC disease could account for the increased mitochondrial cholesterol loading. Hence, we propose that ER stress determines StARD1 upregulation in AD, while in NPC, ACDase downregulation may stand as the trigger to induce StARD1 induction (67). In conclusion, we propose that StARD1 may be crucial for the mitochondrial cholesterol accumulation, characteristic of AD and NPC. Further research is required to determine that targeting this process may be of relevance for both AD and NPC disease.

## Author Contributions

ST, CG-R, and JF-C discussed findings, analyzed literature, wrote the manuscript, and designed the schematic figures.

### Conflict of Interest

The authors declare that the research was conducted in the absence of any commercial or financial relationships that could be construed as a potential conflict of interest.
